# Antibody dependent enhancement infection of Enterovirus 71 *in vitro *and *in vivo*

**DOI:** 10.1186/1743-422X-8-106

**Published:** 2011-03-08

**Authors:** Jian-Feng Han, Rui-Yuan Cao, Yong-Qiang Deng, Xue Tian, Tao Jiang, E-De Qin, Cheng-Feng Qin

**Affiliations:** 1 State Key Laboratory of Pathogen and Biosecurity, Beijing Institute of Microbiology and Epidemiology, Beijing 100071, PR China

## Abstract

**Background:**

Human enterovirus 71 (EV71) has emerged as a significant cause of acute encephalitis and deaths in young children. The clinical manifestations caused by EV71 varied from mild hand, foot and mouth disease to severe neurological complications and deaths, but its pathogenesis remains elusive. Antibody dependent enhancement (ADE) infection has been reported in various viruses and has been shown to contribute to disease severity.

**Results:**

In this study, the presence of sub-neutralizing antibody was demonstrated to enhance EV71 infection in THP-1 cells and increase the mortality of EV71 infection in a suckling mouse model. Further, a secondary infection model was established to characterize the correlation between ADE and disease severity, and primary asymptomatic EV71 infection was shown to increase the mortality of the secondary EV71 infection in suckling mice.

**Conclusions:**

Together, these *in vitro *and *in vivo *experiments strongly supported the hypothesis of ADE infection of EV71. The present findings indicate ADE might contribute to the pathogenesis of severe EV71 infection, and raise practical issues of vaccine development and antibody-based therapy.

## Background

Hand, foot and mouth diseases (HFMD) are common self-limiting illness in infants and young children, characterized by ulcerating vesicles in the mouth and lesions on the hands and feet. Small outbreaks of mild HFMD have occurred periodically throughout the world for a long time. Two closely related viruses, coxsackievirus 16 (CA16) and enterovirus 71 (EV71) have been identified as the most frequent pathogens of HFMD, and other enteroviruses, including CA5 and CA10, can also cause HFMD. While since 1997, large outbreaks of HFMD associated with severe neurological complications and a high case-fatality rate have been reported in Malaysia [1], Taiwan [2], Singapore [3], Japan [4] and other Asian-Pacific areas. In mainland China, large outbreaks of HFMD have been reported since 2008, resulting in millions of cases and hundreds of deaths in children [5]. These severe forms of HFMD have been associated with EV71 infection, which has emerged as an important public health problem.

EV71 is a small, non-enveloped virus with a single positive-stranded RNA genome size of about 7.4 kb in length. It belongs to the family *Picornaviridae*, genus *Enterovirus *together with CA16. Its open reading frame encodes a polyprotein, flanked by 5' and 3' untranslated regions (UTRs). The polyprotein can be further processed into four capsid proteins (VP1, VP2, VP3 and VP4) and seven nonstructural proteins (2A, 2B and 2C, 3A, 3B, 3C and 3D). The capsid protein VP1 is variable and confers distinct antigenic properties. Based on VP1 gene sequence, EV71 can be divided into genotype A, B, and C [6]. Genogroups B and C can be further divided into 5 additional subgenogroups, designated B1-B5 and C1-C5, respectively [4,7]. The predominant genotypes currently circulating are C1, C4, C5 and B5, and different genotypes of EV71 stains may co-circulate in the same areas. Additionally, recombination and positive selection contribute to the antigenic diversity of EV71, and intra- or inter-genotypic recombinant EV71 strains have been reported in large outbreaks in different countries [8,9].

There is currently no specific antiviral therapy to cure and no vaccine to prevent severe EV71 infection, due in part to the lack of understanding of viral pathogenesis. Actually, the clinical manifestations of EV71 infections varied. Most EV71 infections are asymptomatic or limited to mild HFMD and herpangina. However, EV71 is a highly neurotropic virus that can cause severe neurological diseases and complication, such as aseptic meningitis, brainstem encephalitis, acute flaccid paralysis and neurogenic pulmonary edema, which has been reviewed previously [10]. The pathogenesis of severe EV71 infection remains somewhat unclear. Radiologic and pathologic evidences indicated brainstem as the major target of EV71 infection [11]. Study in mice also demonstrated that retrograde axonal transport in neuron cells might be the major transmission route of EV71 [12]. Laboratory and clinical data demonstrated that inflammatory and immune responses also contribute to the pathogenesis of EV71 related severe diseases [13].

Antibody-dependent enhancement (ADE) of virus infection is a phenomenon in which preexisting sub-neutralizing antibodies enhance virus entry and replication. This phenomenon was first described by Hawkes in 1964 [14], and then ADE infection has been identified for many important viruses, including dengue virus, respiratory syncytial virus, human immunodeficiency virus, and Ebola virus. Several reports indicated that ADE was observed for members of the Picornaviridae family including foot-and-mouth disease virus [15], poliovirus [16], and coxsackievirus B [17,18]. Just during our submission, a group from Taiwan successfully demonstrated the ADE infection of EV71 in THP-1 cells for the first time [19].

Seroepidemiological survey has demonstrated that the high morbidity and mortality occur in 6-11 months old infants [20], which is correlated with the coincident decline in maternal antibodies [21]. The association of pre-existing antibodies with the increased severity of disease deserves further concerns and investigation. Here in this study, the possible role of sub-neutralizing antibodies during EV71 infection *in vitro *and *in vivo *was observed, and the association of ADE with disease development was analyzed in a secondary EV71 infection model.

## Results

**Figure 1:**
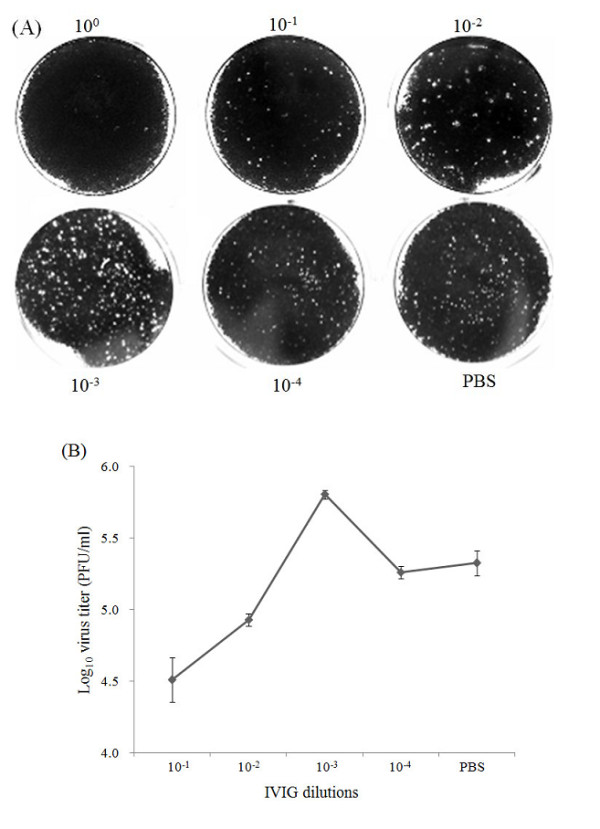
**ADE infection of EV71 in THP-1 cells mediated by different concentrations of IVIG**. Serial 10-fold IVIG dilutions were pre-mixed with EV71 and then cultured in THP-1 cell for 24 h. (A) Plaque forming assay of viral yield in THP-1 cells at 24 h post infection. 10^0 ^dilutions of IVIG treatment completely block EV71 replication in THP-1 cells, an enhancement of viral yield was observed at 10^-3 ^dilutions compared with the PBS control and 10^-2 ^or 10^-4 ^dilutions. (B) The dose range of enhancement of EV71 infection by IVIG. The error bars represent the standard deviations of three independent experiments.

Previously, we have shown that commercial human IVIG preparations manufactured from Chinese plasma donors contain high titer neutralizing antibodies against EV71 [22]. Here to observe the possible ADE effects of EV71 infection, varying concentrations of IVIG preparations were used to incubate with EV71 to form antibody-virus complex before infecting THP-1 cells. The results of plaque assay showed that stock solution of IVIG can completely block EV71 infection in THP-1 cells, and PBS control has no effects on viral yield as expected (Figure 1A). Further dilutions of IVIG decreased the ability of EV71 neutralization, while at the 10^-3 ^dilution of IVIG (50 μg/ml), a significant increase in virus titer was observed. The curves of viral yield from each dilutions coincided well with the ADE phenomenon (Figure 1B). Furthermore, real-time RT-PCR results also demonstrated that high concentration of IVIG (10^-1 ^dilution) prevent EV71 infection, while lower concentration of IVIG (10^-3 ^dilution) enhanced EV71 infection (data not shown). These experiments demonstrated lower concentration of neutralizing antibodies against EV71 can enhance EV71 infection in a monocytic cell line.

**Figure 2:**
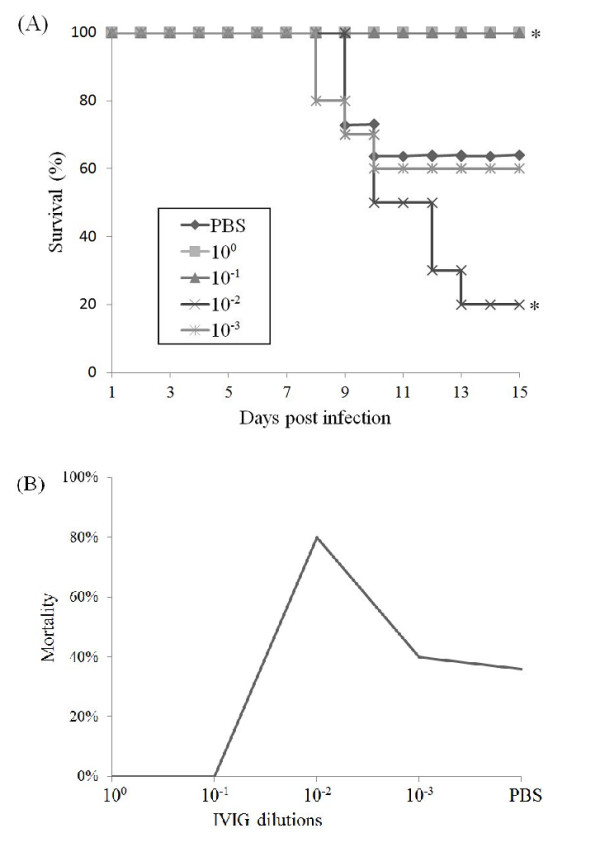
**Lower concentration of IVIG enhanced the mortality of EV71-infected mice**. Groups of 1-day-old mice (n = 11) were injected with 1 LD_50 _of AH08/06 strain pre-mixed with varying concentration of IVIG. The mortality was further monitored for 2 weeks. (A) Kaplan-Meier survival curves were analyzed by the log-rank test and compared to curves of the PBS controls. Significant differences are indicated by asterisks. (B) Mortality curves of mice treated with different IVIG dilutions. 10^-2 ^dilution of IVIG resulted in 80% death in EV71-infected mice.

Next, to characterize the role of IVIG on EV71 infection in vivo, EV71 was pre-mixed with different concentration of IVIG or PBS were intraperitoneally injected into 1-day-old mice (n = 11), respectively. The survival curves (Figure 2A) showed that in the PBS control group, 7 of 11 (64%) mice infected with EV71 infection survived. High concentration of IVIG (10^0 ^and 10^-1 ^dilutions) provided full protection against lethal EV71 challenge, demonstrating the protective role of neutralizing antibodies. Further dilution of IVIG (10^-3 ^dilution) led to a similar effect as the PBS control, indicating no effective neutralizing antibodies at this concentration. Significantly, 10^-2 ^dilution of IVIG (500 μg/ml) increased the mortality of EV71 infection, resulting in 80% deaths in EV71-infected mice. These findings demonstrated that high concentration of IVIG (10^0 ^and 10^-1 ^dilution) can prevent deaths caused by EV71 infection in mice, while the pre-incubation of EV71 with sub-neutralization concentration of IVIG (10^-2 ^dilution) increased the mortality of EV71 infection in mice, thus ADE phenomenon occurred *in vivo *(Figure 2B).

**Figure 3:**
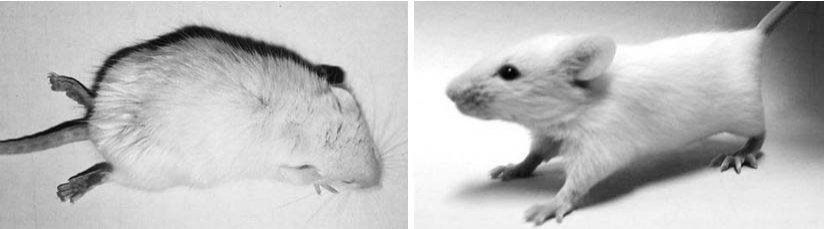
**A representative picture of hind limb paralysis caused by the secondary infection of EV71 (10 days post the secondary infection)**. The mouse on the right-hand side is an age-matched (23-day-old) control.

**Figure 4:**
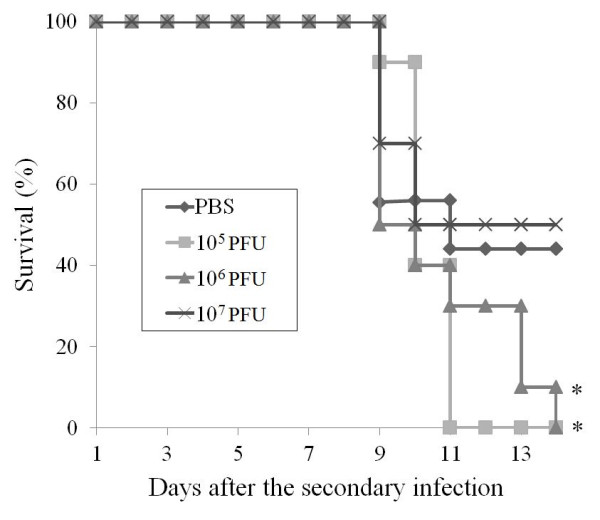
**The secondary infection model of EV71**. Groups of 1-day-old mice were first injected with different doses (10^5^, 10^6^, and 10^7 ^PFU/per mouse) of an avirulent EV71 strain (HN08/08) and then challenged with a virulent EV71 strain (AH08/06) 14 days later. The surviving curves after the secondary EV71 infection was analyzed by log-rank test and significant differences are indicated by asterisks. One representation of three independent experiments is shown.

Secondary infection model of dengue virus had been well established for ADE research in mice [23] and Rhesus monkeys [24]. Here, we manage to develop a secondary infection model of EV71 in which 1-day-old mice were first infected with an avirulent EV71 strain HN08/08 and subsequently infected with a virulent EV71 strain AH08/06 14 days after the primary infection. After the primary infection with HN08/08, all the infected mice survived, and no neurological symptoms developed. Serum examination showed that no neutralizing antibodies (< 1:8) were detected in all of the infected mice, that means only sub-neutralizing antibody was induced. While after the secondary infection with the virulent strain AH08/06, groups of mice that primary injected with HN08/08 developed typical neurological manifestation, including movement disorientation, hind limb paralysis and opisthotonus (Figure 3). The survival curves (Figure 4) showed that 44% (4 of 9) of the mice that primary injected with PBS survived after the secondary infection with AH08/06. While primary injected with 10^5 ^and 10^6 ^PFU of HN08/08 significantly increased the mortality to 100%, and all of the mice died within 9-14 days after the secondary infection. Additionally, primary injected with a higher dose (10^7 ^PFU) of HN08/08 resulted in 50% (5 of 10) deaths, conferring no significant difference with the PBS control group. Thus, previous infection with 10^5 ^and 10^6 ^PFU EV71 was shown to increase the mortality of sequential EV71 infection in mice. Together, these results strongly indicated that the pre-infection induced sub-neutralizing antibodies might enhance EV71 infection, and might be associated with disease development.

## Discussion

Our results provided evidence that pre-existing antibodies can enhance EV71 infection *in vitro *and *in vivo*. High concentration of IVIG can neutralize EV71, while further dilutions of IVIG led to an increase of viral replication and yield in THP-1 cells, and resulted in a higher mortality in mice. In the secondary EV71 infection model, primary infection with an avirulent EV71 strain result in a higher mortality of sequential EV71 infection in mice, indicating previous EV71 infection may be a potential risk factor for severe diseases during sequential EV71 infection. The present findings not only indicate ADE contribute to the pathogenesis of severe EV71 infection, but also raise practical issues about the development of vaccines and passive therapy with antibodies against EV71.

Mouse and cynomolgus macaque models have been established to study the pathogenesis and evaluate antivirals and vaccines [25]. Experimental infection with EV71 has been shown to cause death in neonatal mice in an age- and dose-dependent manner [26]. And 14-day-old suckling mice are susceptible to virulent EV71 strain and have been used for vaccine evaluation [27]. The virulent strain AH08/06 and avirulent strain HN08/08 were both isolated in our laboratory and intraperitoneally injection with AH08/06 can result in neurological symptoms and deaths in different age of suckling mice [28]. EV71 antigen can be detected in the brain, and histopathological analysis of tissues from mice showed signs of infection (unpublished data). By using these strains, the secondary EV71 infection model were developed, and the results showed that preliminary exposure to an avirulent EV71 strain before secondary EV71 infection increased the risk of developing severe neurological complications and deaths. Multiple genotypes of EV71 strains co-circulate in in the same areas for a long time [10], and large outbreaks are probably associated with genotype replacement [8]. A recent report has shown that repeated EV71 infection is common during large outbreaks in China [29]. This is particularly important considering the fact that most primary EV71 infections are asymptomatic.

The target cells of EV71 in human tissues and organs have not been identified. Previous experiments showed that coxsackieviruses could replicate in lymphocytes and monocytes [17,30]. Human monocytic cell line THP-1 has Fc and complement receptor that is used for in vitro ADE assay in this study. The mechanism for ADE of EV71 infection is still unknown. Different mechanisms for ADE has been hypothesized, in which Fc receptor, complement receptor, β2-microglobulin have been reported to play a role. Wang et al recently demonstrated that Fcγ-receptor may participate in EV71-induced ADE based on results from THP-1 cells [19]. Arita et al also reported high-affinity Fc receptor mediated enhancement of poliovirus infection [31]. Further, EV71 encodes 4 structural proteins (VP1-4), and the viral antigenic determinants associated with ADE of EV71 are also critical for future investigation. Recently, Chehadeh et al. [32] demonstrated that VP4 protein is the major target of enhancing antibodies in the CVB4 and CVB3 ADE model. By using monoclonal antibodies screening, the prM protein of dengue virus was recently identified as the critical ADE determinants [33]. We have produced a panel of EV71 mAbs and such efforts are currently underway. Additionally, ADE infection of non-enveloped virus requires binding of virus-antibody complexes both to Fcγ-receptor and to the specific viral receptor [34]. The role of PSGL-1 [35] and SCARB2 [36], two EV71 receptors, during ADE of EV71 infection deserves further research.

IVIG is a pharmaceutically preparation of human IgG that has immunoregulatory and anti-inflammatory properties. Clinically administration during EV71 outbreaks in Taiwan [37,38] had achieved an improvement in clinical outcome. The Ministry of Health of China has recommended high dose (total 2 g/kg in 2-5 days) of IVIG for treatment of severe EV71 infection. However, considering the fact that IVIG contains anti-EV71 antibodies [22], the potential risk of IVIG induced ADE of EV71 infection, especially the dose, must be carefully considered in future clinical application. The enhancing concentrations of IVIG may vary. Our experiments showed 50 μg/ml of IVIG enhanced EV71 infection *in vitro*, while 500 μg/ml *in vivo*. Wang et al showed that 0.5-1 or 8-16 mg/ml of IVIG enhanced EV71 infection in THP-1 cells. The difference may results from the different sources of IVIG and the methodology applied [19,22].

Given the public health significance of EV71, a wide range of experimental EV71 vaccine approaches have been studied, and neutralizing antibodies have been suggested as one of the most important factors in limiting the severity of infection. But considering the possible impact of ADE, vaccines should be carefully designed to avoid the induction of known enhancing antibodies. Antibody responses induced by inactivated or subunit vaccines tend to be associated with severe disease by respiratory syncytial virus and retroviruses [39,40]. Induction of cellular immunity, rather than antibodies, may represent an alternative strategy for future EV71 vaccine development [41]. Live attenuated vaccine other than inactivated vaccine will be a good candidate.

Taken together, the present findings from laboratory and animal experiments demonstrate that the presence of sub-neutralizing antibodies would enhance EV71 infection, and primary EV71 infection might represent a risk factor of severe diseases during sequential EV71 infection. Controlled clinical trials are critically needed to confirm the correlation between disease severity and ADE. Also, much effort is currently underway to determine the cellular and viral determinants of EV71-induced ADE. The results of this and future studies thus contribute to a better understanding of the pathogenesis of EV71 infection, as well as an improved ability to evaluate vaccines and therapeutic antibodies.

## Materials and methods

### Viruses and cells

Human rhabdomyosarcoma (RD) cells were cultured with DMEM supplemented with 2% fetal bovine serum (FBS), 100 IU of penicillin, and 100 μg of streptomycin per ml at 37°C in the presence of 5% CO_2_. Human monocytic cells (THP-1) were used for *in vitro *ADE assay and maintained in RPMI-1640 media at 37°C in 5% CO_2_. EV71 strain AH08/06 was isolated from the throat swab sample of an HFMD case during an outbreak in 2008 in Anhui, China [39]. EV71 strain HN08/08 was isolated from the stool sample of an HFMD case during an outbreak in 2008 in Henan, China. All the viruses were propagated and titrated in RD cells and the stock was stored in aliquots at -80°C in our laboratory. The titer of EV71 was expressed as 50% tissue culture infection dose (TCID_50_), plaque-forming units (PFU) or 50% lethal dose (LD_50_) according to the Reed-Muench method [42].

### Microneutralizing assay

The titer of neutralizing antibodies against EV71 was determined in RD cells according to standard protocol [43]. Briefly, 50 μL of sample dilutions and 50 μL of virus stock containing 100 TCID_50 _EV71 were mixed and incubated onto the microtiter plates with RD cells at 37°C for 6 days. All the samples were tested at an initial dilution of 1: 8, and cell and virus controls were run simultaneously. The neutralizing antibody titer was defined as the highest dilution of serum that could prevent the occurrence of cytopathic effect.

### Human Intravenous Immunoglobulin (IVIG) preparations

Human 5% liquid IVIG preparations (5 g/100 ml, Tonrol Bio-Pharmaceutical) manufactured from Chinese plasma donors, consisting more than 99% of IgG and a very small quantities of IgA and IgM, were used as stock dilutions. The titer of neutralizing antibodies of IVIG was calculated to 1:128 by microneutralzing assay as previously described.

### ADE infection assay in vitro

Stock IVIG preparation (10^0 ^dilution) was 10-fold serially diluted to 10^-4 ^with PBS and each dilution was then incubated with EV71 suspensions at 37°C for 1 h to form virus-antibody complexes, respectively. The EV71-antibody complexes were then added to cultured THP-1 cells at a multiplicity of infection (MOI) of 1 and incubated for 1 h at 37°C. Then, THP-1 cells were washed for three times with PBS and then incubated with fresh RPMI-1640 media for another 24 h. Finally, the infected cells and supernatants were harvested and the virus yield was determined by the plaque assays and real-time RT-PCR assays.

### Plaque forming assay

Monolayer RD cells were cultured in 12-well plates and serial 10-fold dilutions of viral suspensions were added for adsorption for 1 h. Then, the virus suspension was replaced with DMEM containing 2% FBS and low melting point agarose. Followed by incubation for 72 h, the cells were fixed with 10% formaldehyde and subsequently stained with 1% crystal violet solution. The titer of EV71 was calculated and expressed as plaque-forming units (PFU)/ml.

### Real-time RT-PCR assay

Viral RNA in EV71-infected THP-1 cells was assayed by real-time RT-PCR as previously described [44]. Viral RNA was extracted by the QIAmp Viral RNA Mini kit (Qiagen) and one-step RT-PCR was performed by using the PrimeScript One-Step RT-PCR kit (Takara) according to the manufacturer's instructions in Roche LightCycler 2.0 systems.

### In vivo neutralization assay of EV71

Stock IVIG preparation was 10-fold serially diluted mixed with EV71 suspensions as previous described. Four groups of 1-day-old Kunming mice (n = 11) were injected intraperitoneally with these EV71-IVIG or EV71-PBS mixtures, respectively. The mortality and neurological symptoms were monitored for 15 days.

### Secondary EV71 infection model

We use two EV71 strains to establish the secondary EV71 infection model: a virulent strain AH08/06 which can result in death in 14-day-old suckling mice and an avirulent strain HN08/08. Briefly, groups of suckling mice of 1-day-old were primary injected subcutaneously with 50 μl of different doses (10^5^, 10^6 ^and 10^7 ^PFU, respectively) of HN08/08 or PBS. Then 14 days after the primary infection, all the mice were subsequently infected with 1 LD_50 _(50 μl) of AH08/06 by intraperitoneally injection. Before the secondary infection, 100 μl of blood was obtained via tail vein bleeding and processed for microneutralizing assay. The mortality and neurological symptoms were followed for another 14 days post the secondary infection. All the animal experiments were approved and performed according to the guideline of Animal Experiment Committee of State Key Laboratory of Pathogen and Biosecurity.

### Statistical analysis

Kaplan-Meier survival curves were used to display mortality data, and log rank analyses were performed to determine statistical significance between different groups.

## List of Abbreviations

ADE: Antibody dependent enhancement; EV71: Enterovirus 71; HFMD: Hand, foot and mouth disease; IVIG: Intravenous Immunoglobulin; MOI: Multiplicity of infection; PFU: plaque-forming units; TCID_50_: 50% tissue culture infection dose.

## Competing interests

The authors declare that they have no competing interests.

## Authors' contributions

JFH and RYC performed the experiments and drafted the manuscript. YQD, TJ and XT participated in cell culture and animal experiments. EDQ and CFQ designed the study, supervised the work and edited the final version of this manuscript. All authors read and approved the final manuscript.
